# Development and Validation of the Perceptions of Research Trustworthiness Scale to Measure Trust Among Minoritized Racial and Ethnic Groups in Biomedical Research in the US

**DOI:** 10.1001/jamanetworkopen.2022.48812

**Published:** 2022-12-29

**Authors:** Sarah C. Stallings, Jennifer Cunningham-Erves, Carleigh Frazier, Jabári S. Ichimura, Thelma C. Hurd, Jordan Jurinsky, Amber Acquaye, Jacquelyn S. Dalton, Consuelo H. Wilkins

**Affiliations:** 1 Division of Geriatric Medicine, Department of Medicine, Vanderbilt University Medical Center, Nashville, Tennessee; 2 Meharry-Vanderbilt Alliance, Nashville, Tennessee; 3 Department of Internal Medicine, Meharry Medical College, Nashville, Tennessee; 4 Public Health Department, University of California, Merced, Visalia; 5 Department of Surgery, University of Texas Health Science Center, San Antonio; 6 Department of Human and Organizational Development, Peabody College of Education and Human Development, Vanderbilt University, Nashville, Tennessee

## Abstract

**Importance:**

Historically, trust in biomedical research has been lower among minoritized racial and ethnic groups who are underrepresented in and excluded from research, with the same groups experiencing worse health outcomes. Unfortunately, instruments that measure trust may not capture components of trust relevant to minoritized racial and ethnic groups.

**Objective:**

To develop and validate a scale to measure trust in biomedical research among minoritized racial and ethnic groups.

**Design, Setting, and Participants:**

This cross-sectional, community-based survey study compared trust and distrust in biomedical research among Black, Latino, and White subgroups in the US using the Perceptions of Research Trustworthiness (PoRT) scale. The scale was developed between March 22, 2016, and September 19, 2018, as part of this study, and its structure, reliability, and validity were examined during pilot (n = 381) and validation (n = 532) phases between February 4, 2019, and July 27, 2021. Convenience samples of adult participants (aged ≥18 years) were recruited locally (Nashville, Tennessee, and San Antonio, Texas) and nationally through the ResearchMatch and Cint online platforms.

**Main Outcomes and Measures:**

Overall and individual item Trust and Distrust subscale scores were compared. Overall Trust and Distrust scores were compared by race and ethnicity using a Kruskal-Wallis H test and individual item scores were compared using independent samples *t* test.

**Results:**

Of the 532 participants in the scale validation study, 144 (27.1%) were Black, 90 (16.9%) were Latino, and 282 (53.0%) were White. Participants had a median age of 43 years (range, 18-90 years), 352 (66.2%) were women, and 198 (37.2%) had educational attainment levels less than a college degree. Factor analysis of the 18-item PoRT scale revealed a 2-factor structure with two 9-item PoRT subscales (Trust and Distrust), which demonstrated high internal consistency (Cronbach α = 0.72 and 0.87, respectively). Mean (SD) Trust subscale scores were lower among Black (34.33 [2.02]) and Latino (34.55 [1.97]) participants compared with White participants (36.32 [1.81]; *P* < .001). Mean (SD) Distrust subscale scores were higher among Black (21.0 [2.15]) and Latino (20.53 [2.21]) participants compared with White participants (18.4 [2.03]; *P* < .001). Individual item results showed that Black and Latino participants were less trusting and more distrusting than White individuals on items related to risks, harms, secrecy, confidentiality, and privacy.

**Conclusions and Relevance:**

These findings suggest that the PoRT scale incorporates trust and trustworthiness concepts relevant among Black and Latino individuals and may allow more precise assessment of trust in research among these groups.

## Key Points

**Question:**

Can a tool such as the Perceptions of Research Trustworthiness (PoRT) scale measure trust in biomedical research reliably, and is it valid for use among minoritized racial and ethnic groups?

**Findings:**

In the validation phase of this survey study with 532 US participants, we observed that Black and Latino participants were less trusting and more distrusting of biomedical research compared with White participants. There were no significant differences in trust scores among Black and Latino participants on any individual items or by educational attainment.

**Meaning:**

These findings suggest that the validated PoRT scale allows trust and distrust in research to be measured using constructs that center the perspectives of Black and Latino individuals, which may aid in developing strategies to improve the trustworthiness of research.

## Teaser

This survey study describes the development and validation of the Perceptions of Research Trustworthiness Scale to measure trust in biomedical research among minoritized racial and ethnic groups in the US.

## Introduction

In the US, Black and Latino individuals represent 13.4% and 18.5% of the population^1^ but account for just 5.0% and 1.0% of biomedical research participants, respectively.^2,3,4,5^ Many factors contribute to minoritized racial and ethnic groups being underrepresented in and excluded from research, including not being invited to participate.^6,7,8,9,10,11,12,13,14^ Lack of trust in research and concerns about the trustworthiness of researchers and research institutions are commonly cited barriers to enrolling in research studies. However, trust and trustworthiness contribute more broadly to research challenges, including community engagement in research design and implementation and downstream issues such as public confidence in research findings.^5,12,15,16,17,18^

Systematic reviews of trust measures have identified concerns with validity and reliability, relevance across topics and over time, and limited inclusion of content areas of trust.^19,20,21,22^ Dimensions of trust consistently confirmed among White individuals include honesty, competency, fidelity, confidentiality, and global and/or system trust.^20^ However, people who identify as Black and/or Latino more often relate trust to safety, fairness, communication, and honesty.^17,23,24,25^ These differences, possibly subtle, may reflect perceptions of trustworthiness in medicine and science that come from personal and group experiences with health systems, access to health care, experiences with discrimination and racism, and unethical biomedical research in minoritized racial and ethnic groups.^26,27,28^ Concerns about fairness, safety, and community benefit may reflect the spillover of individual and group experiences often associated with structural racism,^29,30,31^ such as negative encounters with police, which have been associated with medical mistrust and have a spillover effect on the mental health of racialized groups.^32,33^ Black and Latino participants’ perceptions of trust and trustworthiness may also reflect distrust related to racist and deceptive research practices, which have exploited and dehumanized minoritized racial and ethnic groups.^17,34,35^

Effectively measuring trust in biomedical research requires instruments that include content areas reflecting minoritized racial and ethnic populations’ definitions and perceptions of trust. We believe that an approach that engages groups known to have lower levels of trust will result in a measurement tool that more accurately reflects trust across populations. Few scale development studies have thoroughly examined the factors that contribute to trust and distrust in biomedical research, especially among Black and Latino populations^20,36^ and among populations with limited educational attainment or limited health literacy,^22^ who are often less trusting.

In this study, we developed and validated a tool to measure trust in biomedical research that is relevant and validated across Black, Latino, and White communities, including those with lower educational attainment. The goal is to more accurately measure trust and subsequently enable strategies aimed at increasing trust and trustworthiness to be evaluated and monitored.

## Methods

The components of this survey study were approved by the Vanderbilt University Institutional Review Board. Informed consent was obtained from all participants prior to their involvement. Respondents were compensated for their participation. Data are reported based on the American Association of Public Opinion Research (AAPOR) Transparency Initiative reporting guideline.

We used a 4-stage process to develop a scale to measure trust in biomedical research: (1) content and item generation, (2) item evaluation through mapping and cognitive interviews, (3) pilot testing, and (4) scale validation. Each stage involved recruitment and sampling procedures most appropriate for the study in that stage, and the validation study is reported in detail here. Stages 1 and 2 were conducted between March 22, 2016, and September 19, 2018; stages 3 and 4 were completed between February 4, 2019, and July 27, 2021. Additional details for all stages can be found in Supplement 1.

Our approach was informed by Cross-Cultural Survey Guidelines,^37^ a resource intended to maximize cross-cultural comparability and minimize measurement and representation errors, and included forming a diverse transdisciplinary research team comprising members with varied research expertise and relevant cultural backgrounds. Expertise of team members included scale development and psychometrics, survey design, public health, community engagement, racial and ethnic health disparities, clinical care, and clinical trials.

### Stage 1: Content and Item Generation

To generate scale content, we used a multistep process that included developing a literature-based conceptual framework, conducting focus groups, and iteratively choosing items using feedback of the research team. Items from 3 existing scales comprised the initial item bank, including 2 trust scales specific to biomedical research^38,39^ and the Distrust Index, which includes 7 items on trust of physicians, 4 of which are focused on research.^15^

We conducted seven 90-minute focus groups with Black, Latino, and White groups exploring perceptions and determinants of trust, privacy, confidentiality, and research participation (published in full elsewhere^40^). Qualitative analysis suggested differences by group in conceptions of research and trust in research.^40^ Three themes emerged: (1) who is trustworthy as a medical researcher, (2) what influences perceptions of trustworthiness in medical research, and (3) what institutions or settings are trustworthy for medical research. Black and Latino participants identified risks, harms, privacy, secrecy, community benefit, and profit incentives as concepts related to trust and trustworthiness in medical research. We supplemented the literature-based conceptual framework with these additional items (eFigure and eTable 1 in Supplement 1).

### Stage 2: Scale Item Evaluation

Stage 1 yielded a pool of 42 items. A panel of researchers mapped each item to 1 or more trust dimensions. Items were removed if redundant or for lack of fit (eg, “research is morally wrong”), were retained if they reflected trust dimensions not covered in previous instruments, or were remapped to a different trust dimension. A consensus was reached on a pilot scale, which included 19 items.

We conducted cognitive interviews to evaluate possible sources of response error based on lack of clarity in the item as worded.^41,42^ Two groups of 9 community members from Nashville, Tennessee, conveyed feedback on items using a “think-aloud” approach to share how they understood items and whether each was clear. Items were revised based on analysis of the cognitive interviews.

### Stage 3: Pilot Study

The initial 19-item scale was tested with 381 individuals with a near-equal distribution of Black, Latino, and White participants (Table 1). The scale was part of a 42-item survey that included questions on demographic characteristics, health care use, and prior participation in medical research. Participants were recruited in person via collaboration with community partners in Nashville and San Antonio, Texas, and online through 2 platforms: ResearchMatch and Cint.^43,44^ For participants recruited in person, the survey was offered on a tablet and a paper survey was available. Online survey responses were collected using REDCap (Research Electronic Data Capture), and paper survey responses were entered by a research team member into REDCap.^45^ Additional method details for the pilot study are included in Supplement 1.

**Table 1.** Self-reported Demographics for Pilot and Validation Survey Participants

Characteristic	No. of participants (%)	
	Pilot study (n = 381)	Validation study (n = 532)
Age, y, median (range)	44 (19-87)	43 (18-90)
Race and ethnicity (self-reported)		
American Indian or Native American	1 (0.3)	2 (0.4)
Asian or Asian American	1 (0.3)	3 (0.6)
Black, African American, or African	118 (31.0)	144 (27.1)
Hispanic, Latino, or Spanish	94 (24.7)	90 (16.9)
Middle Eastern or North African	0	2 (0.4)
Native Hawaiian or Pacific Islander	1 (0.3)	0
White	136 (35.7)	282 (53.0)
Multiracial^a^	13 (3.4)	7 (1.3)
Missing	15 (3.9)	2 (0.4)
Education		
Less than a college degree	189 (49.6)	198 (37.2)
College degree or higher	190 (49.9)	317 (59.6)
Missing	2 (0.5)	17 (3.2)
Gender identity		
Men	160 (42.0)	167 (31.4)
Women	218 (57.2)	352 (66.2)
Nonbinary	1 (0.3)	8 (1.5)
Transgender	0	3 (0.6)
Missing	2 (0.5)	2 (0.4)
Household income, $		
0-24 999	137 (35.9)	75 (14.1)
25 000-49 999	95 (24.9)	121 (22.8)
50 000-99 999	98 (25.7)	203 (38.1)
≥100 000	50 (13.1)	133 (25.0)
Missing	1 (0.3)	0
Health insurance status		
Insured	322 (84.5)	489 (91.9)
Uninsured	58 (15.2)	33 (6.2)
Missing	1 (0.3)	10 (1.9)

^a^ Includes participants who checked more than 1 race and ethnicity option on the survey.

To identify latent factors, exploratory factor analysis was conducted on the 19-item scale. Results were reviewed by research team members to ensure congruency with past findings and trust domain coverage. One item was deemed ambiguous by exploratory factor analysis and unclear in cognitive interviews (Supplement 1). This item (“People in medical research studies get better health care than people who aren’t in research studies”) was removed, and the resulting 18-item Perceptions of Research Trustworthiness (PoRT) scale was tested in the validation study.

### Stage 4: Validation Study Methods

For the validation study, the 18-item PoRT scale was included in a 69-item survey that contained the same demographic, health care use, and medical research participation variables as in the pilot study, plus validated scales for trust in health care organizations and professionals, health literacy, numeracy, and social desirability. Two weeks after completing this survey, respondents were presented with a retest of the PoRT scale to measure test-retest reliability.

Participants were recruited from a national pool of adults (aged ≥18 years) through 2 different online platforms: ResearchMatch and Cint.^43,44^ Because both platforms allow survey invitations to be sent based on race and ethnicity, invitations were targeted to recruit at least 100 individuals of each group (Black, Latino, and White). Respondent demographic characteristics were closely monitored throughout recruitment to allow targeting of invitation waves to less represented populations. Data were collected from November 4, 2019, to September 29, 2020.

Participants self-reported race and ethnicity in response to a single question with the following options: American Indian or Native American; Asian or Asian American; Black, African American, or African; Hispanic, Latino, or Spanish; Middle Eastern or North African; Native Hawaiian or Pacific Islander; or White. Participants could select multiple options. Throughout, we refer to participants who selected “Black, African American, or African” as Black and participants who selected “Hispanic, Latino, or Spanish” as Latino.

### Statistical Analysis of Validation Study Data

We used descriptive statistics to summarize responses to demographic questions and contextual questions (eg, previous participation in research). We reported the mean (SD) scores of the overall trust scale and the subscales Trust and Distrust. Item scores reported are mean (SD) score per item. We used Pearson correlation coefficients to assess correlations between various trust dimensions in the item mapping stage. Exploratory factor analysis using principal component extraction with an oblimin rotation was performed to determine scale and subscale structure. A factor loading above the 0.40 cutoff point along with visual examination of the scree plot was used to determine the number of factors to retain.

The factor analysis of PoRT scale validation data confirmed a 2-subscale structure as seen in the pilot data; Kaiser-Meyer-Olkin (KMO) and Bartlett tests evaluated adequacy of sampling and significance. For internal consistency analysis, we used (1) Cronbach α and item-total correlations (ITCs) for the 2 subscales and (2) Cronbach α item deleted and ITC for individual scale items. Spearman’s ρ correlation coefficients were used to examine the associations between Trust and Distrust subscale scores and scores on other measures (eg, health literacy, health numeracy, Marlowe-Crowne Social Desirability Scale score, trust in health care organizations and professionals, and willingness to participate in research, as well as background, health status, and health care use measures). Test-retest reliability was assessed with the following: nonparametric interclass and intraclass correlations, criterion validity of various factors (education, numeracy, and literacy) determined by Spearman rank-order correlation, nonparametric means difference Kruskal-Wallis *H* test for known-groups validity (Black, Latino, or White), and the independent-samples *t* test for differences in item ratings by race and ethnicity (eTable 3 in Supplement 1 provides additional detail, including mean score coding ranges for variables). We calculated the response rate for the overall sample based on surveys with a minimum of 75.0% of questions answered among those who received the survey.

All *P* values were 2-sided, and *P* < .05 was set as the threshold for statistical significance. Data were analyzed using SPSS, version 26.0 (IBM SPSS Statistics) between December 6, 2020, to July 27, 2021.

## Results

Of the 532 participants in the validation study, 144 (27.1%) were Black, 90 (16.9%) were Latino, and 282 (53.0%) were White. Their median age was 43 years (range, 18-90 years). With regard to gender identity, 352 participants (66.2%) identified as women, 167 (31.4%) as men, 8 (1.5%) as nonbinary, and 3 (0.6%) as transgender. Additional demographic data are presented in Table 1. Respondents resided in 45 states and the District of Columbia. States with the most respondents were New York (53 [10.0%]), California (42 [7.9%]), Ohio (40 [7.5%]), Texas (36 [6.8%]), Tennessee (29 [5.5%]), Georgia (25 [4.7%]), and Florida (25 [4.7%]); there were no respondents from Maine, Montana, Nebraska, Rhode Island, or Wyoming. The survey response rate for online invitations was 43.0%.

Factor analysis of the final 18-item PoRT scale confirmed a 2-factor scale with a Trust subscale (9 items; Cronbach α = 0.72) and a Distrust subscale (9 items; Cronbach α = 0.87). In test-retest analysis, reliability was 0.75 (*P* < .001) in the Trust subscale and 0.78 (*P* < .001) in the Distrust subscale. Interclass correlations for the Trust and Distrust subscales were high (*r*_s_ = 0.71 and 0.76, respectively) as were intraclass correlations (*r*_s_ = 0.75 and 0.86, respectively). Additional psychometric analysis of the scale including KMO and Bartlett tests, Cronbach α if item omitted, and ITCs can be found in Table 2 and eTable 2 in Supplement 1.

**Table 2.** Perceptions of Research Trustworthiness Scale Scores by Minoritized Racial and Ethnic Groups

PoRT subscale and trust statement (numbered as in the survey)	Mean (SD) participant score^a^				ITC	Cronbach α if omitted^b^
	All (N = 532)	Black (n = 144)	Latino (n = 90)	White (n = 282)		
Trust						
1. Medical researchers tell people everything they need to know about being in a research study.	3.78 (1.03)	3.67 (1.04)	3.54 (1.17)	3.93 (0.95)	0.47	0.68
3. Any info about me that I give to medical researchers would be kept confidential.	4.23 (0.83)	4.22 (0.86)	4.24 (0.83)	4.24 (0.8)	0.45	0.69
4. Medical researchers would never give someone something that would hurt them, just to study how it works in people.	3.41 (1.23)	3.2 (1.26)	3.36 (1.26)	3.55 (1.19)	0.49	0.68
7. Participation in medical research benefits society.	4.64 (0.55)	4.49 (0.6)	4.61 (0.56)	4.72 (0.5)	0.45	0.70
9. Medical researchers usually tell people in a research study about different things they could do to get well.	3.25 (1.07)	3.23 (1.11)	3.24 (1.11)	3.28 (1.05)	0.18	0.74
10. If I had a chance to be in a medical research study, it would be easy for me to decide to join in or not.	3.58 (1.19)	4.03 (0.98)	4.17 (0.96)	4.19 (0.93)	0.43	0.69
11. Medical researchers only do research on people who know it is happening.	4.14 (0.95)	3.4 (1.24)	3.14 (1.20)	3.82 (1.11)	0.44	0.69
14. My physician would not ask me to be in a medical research study if [they] thought it would hurt me.	4.2 (0.92)	3.93 (1.06)	4.12 (0.95)	4.35 (0.81)	0.39	0.70
18. If I had a chance to be in a medical research study, I would be sure that participating in medical research would be the best choice for me.	4.15 (0.87)	4.16 (0.86)	4.13 (0.82)	4.24 (0.80)	0.36	0.70
Mean Trust subscale score (sum of 9 items)	35.38 (1.94)	34.33 (2.02)	34.55 (1.97)	36.32 (1.81)	NA	NA
Distrust						
2. If I had a chance to be in a medical research study, I wouldn’t be sure about being in medical research or not.	2.34 (1.15)	2.52 (1.25)	2.3 (1.13)	2.25 (1.10)	0.50	0.86
5. Medical researchers keep dangerous things that could happen to people in a medical research study secret.	2.24 (1.13)	2.35 (1.09)	2.47 (1.19)	2.12 (1.13)	0.66	0.85
6. Medical researchers try to hide any mistakes they make in their research studies.	2.45 (1.05)	2.58 (1.04)	2.53 (1.12)	2.37 (1.03)	0.66	0.85
8. Medical research is secretly designed to give diseases to minority groups.	1.58 (0.88)	1.92 (0.92)	1.73 (0.95)	1.36 (0.75)	0.64	0.85
12. Medical researchers would lie to people to convince them to be in a research study.	1.93 (0.98)	2.18 (1.06)	2.07 (1.12)	1.78 (0.88)	0.74	0.84
13. Medical researchers are more interested in helping their own careers than helping people be healthy.	2.12 (0.98)	2.16 (0.92)	2.2 (1.05)	2.07 (0.99)	0.63	0.85
15. It is very likely that I, or people like me, will be used as guinea pigs in medical research.	2.74 (1.27)	2.93 (1.22)	3.01 (1.28)	2.58 (1.28)	0.42	0.87
16. Medical researchers will share my personal info with anybody else they want to, even if I don’t tell them they can do that.	1.75 (0.89)	1.97 (0.95)	1.84 (0.89)	1.6 (0.83)	0.72	0.84
17. I’m not sure that I have a voice in who can use my medical information.	2.32 (1.18)	2.39 (1.23)	2.38 (1.23)	2.27 (1.15)	0.54	0.86
Mean Distrust subscale score (sum of 9 items)	19.47 (2.11)	21 (2.15)	20.53 (2.21)	18.4 (2.03)	NA	NA

Abbreviations: ITC, item-total correlation; NA, not applicable; PoRT, Perceptions of Research Trustworthiness.

^a^ The mean coding ranges for mean scores are provided in eTable 3 in Supplement 1.

^b^ For the Trust and Distrust subscales, Cronbach α was 0.72 and 0.87, respectively.

Mean (SD) scores on the PoRT Trust subscale were lower among Black (34.33 [2.02]) and Latino (34.55 [1.97]) participants compared with White participants (36.32 [1.81]; Kruskal-Wallis *H* = 17.35; *P* < .001). Mean PoRT Distrust subscale scores were higher among Black (21.0 [2.15]) and Latino (20.53 [2.21]) participants compared with White participants (18.4 [2.03]; *H* = 21.60; *P* < .001). Individual item scores for the PoRT scale are shown in Table 2.

Minoritized racial and ethnic group differences were found for 8 individual items, including 5 on the Trust subscale and 3 on the Distrust subscale (Table 3). Compared with White participants, Black participants were less trusting and more distrusting on items related to secrecy, risk and/or harms, safety, community benefit, honesty, fairness, privacy, and confidentiality and Latino participants were less trusting and more distrusting on items related to secrecy, risk and/or harms, safety, and fairness. There were no significant differences among Black and Latino participants on any individual items (eTable 4 in Supplement 1).

**Table 3.** Perceptions of Research Trustworthiness Scale Items Mapped to Trust Dimensions During Development of the Scale^a^

PoRT subscale item	Dimension		Item mean differences by minoritized racial and ethnic group (95% CI)
	Theoretical from literature	Practical from focus groups	
Trust			
1. Medical researchers tell people everything they need to know about being in a research study.	Honesty, communication	Secrecy	Latino < White, −0.36 (−0.65 to −0.08)
4. Medical researchers would never give someone something that would hurt them, just to study how it works in people.	Honesty, fidelity		Black < White, −0.39 (−0.65 to −0.13)
7. Participation in medical research benefits society.		Community benefit	Black < White, −0.25 (−0.37 to −0.13)
11. Medical researchers only do research on people who know it is happening.	Fidelity, safety	Risk and/or harms	Latino < White, −0.59 (−0.88 to −0.30)
14. My physician would not ask me to be in a medical research study if [they] thought it would hurt me.	Confidence, fidelity, safety	Risk and/or harms	Black < White, −0.43 (−0.63 to −0.22)
Distrust			
8. Medical research is secretly designed to give diseases to minority groups.	Fairness, safety, systems trust	Risk and/or harms, secrecy	Latino > White, 0.41 (0.18 to 0.64)
			Black > White, 0.56 (0.38 to 0.75)
12. Medical researchers would lie to people to convince them to be in a research study.	Honesty	NA	Black > White, 0.43 (0.21 to 0.64)
16. Medical researchers will share my personal info with anybody else they want to, even if I don’t tell them they can do that.	Confidentiality	Privacy	Black > White, 0.36 (0.17 to 0.56)

Abbreviations: NA, not applicable; PoRT, Perceptions of Research Trustworthiness.

^a^ We show individual scale items with significant mean differences by race and ethnicity category and the trust dimensions mapped to them during scale development.

There was no difference in either Trust or Distrust subscale scores by gender (men vs women) or education (high school diploma/GED [General Education Development] or less vs any college). Trust scores were positively correlated with literacy scores (Spearman’s rank-order, *r*_s_ = 0.14; *P* < .001) and numeracy scores (*r*_s_ = 0.22; *P* < .001). Distrust scores were negatively correlated with literacy scores (*r*_s_ = −0.29; *P* < .001) and numeracy scores (*r*_s_ = −0.15; *P* < .001).

## Discussion

Trust in biomedical research and the trustworthiness of researchers play a central role in public confidence in research findings and public participation in research, yet development of validated measures of trust with relevance to minoritized racial and ethnic groups is still critically needed. In this survey study, we developed and validated the PoRT scale in a diverse sample and found lower trust and higher distrust among Black and Latino participants compared with White participants. Our cross-cultural approach prioritized inclusion of concepts of trust common among populations that have been historically excluded from and exploited by research and have experienced injustices related to health and science more broadly.^40^

The PoRT scale is an internally consistent and reliable 18-item instrument that measures 2 related and negatively correlated concepts—trust and distrust—in the context of biomedical research. The scale was developed and tested among populations of Black, Latino, and White individuals with varying educational attainment. The PoRT scale includes items that Black and Latino individuals more often relate to trust such as risks and/or harms, fairness, safety, secrecy, and community benefit, which are often missing from existing measures of trust.^17,23,24,25^ Our overall findings are consistent with previous studies of trust and distrust among minoritized racial and ethnic groups who have been historically marginalized.^15,24^

In addition to overall differences among minoritized racial and ethnic groups, we found differences in individual items that suggest Black and Latino people may lack trust or have more distrust for differing reasons, which brings to light shared and distinct factors that influence perceptions of trustworthiness in biomedical research. On 8 individual items (5 on the Trust subscale and 3 on the Distrust subscale) related to secrecy, honesty, fairness, risk and/or harms, safety, community benefit, and privacy, we found differences among Black and White participants on 5 items and among Latino and White participants on 2 items (Table 3). Responses to only 1 item (“Medical research is secretly designed to give diseases to minority groups”) were significantly different for both Black and Latino participants compared with White participants (Table 3), which may reflect well-documented medical exploitations and racism.^34,46^ Differences on 2 specific items suggest that both Black and Latino participants believe researchers are less trustworthy. Latino participants were less likely to agree that “medical researchers tell people everything they need to know about being in a research study,” and Black participants were more likely to agree that “medical researchers would lie to people to convince them to be in a research study.” This supports recent shifts toward expecting researchers and research institutions to demonstrate their trustworthiness instead of placing the burden on minoritized racial and ethnic communities to be more trusting.

We did not observe significant differences among scale and subscale scores of Black and Latino participants, which suggests the high performance of the PoRT scale in capturing aspects of trust that these 2 minoritized racial and ethnic groups share. This may also point to an area where further research could find distinctions between the groups.

### Limitations

Findings from this survey study are limited by response bias, as those responding to the invitations to participate may be positively predisposed to engage in research. However, we recruited some participants in person in community settings to increase involvement of individuals who might not typically volunteer for research. Our study focused on the largest minoritized racial and ethnic groups in the US, and our recruitment and sampling methods used strategies to include individuals with limited educational attainment. Nonetheless, our findings may not be generalizable to other groups such as Asian individuals, Indigenous individuals, Native Hawaiian or Pacific Islander individuals, sexual and gender minorities, or other historically marginalized groups. Trust remains understudied among Asian, Asian American, and Indigenous populations despite ongoing marginalization and recent increases in anti-Asian racism.^47,48,49,50,51,52^ Our scale does not specifically capture concepts related to colonization, respect for cultural beliefs, and experiences with microaggressions, which may be relevant to trust among Indigenous and Asian groups.^52,53,54^

Although our sample was racially, ethnically, and educationally diverse, health insurance rates were high, especially in the validation sample. Most of our participants were women, which is similar to many survey-based studies; however, trust in research may be different among Black and Latino men, who are less likely to seek health care and participate in research.^55,56,57^ Finally, the first 3 stages of our data collection were completed prior to the COVID-19 pandemic and the validation study was completed early in the pandemic. The pandemic magnified long-standing inequities in health care access, ineffective public health communication tactics, mistrust of research results, and hesitancy to adopt evidence generated in research. Perspectives on trust and trustworthiness may have shifted during the pandemic and the co-occurring heightened awareness of racial injustices.^58^

## Conclusions

There is a critical need to assess and better understand trust in research among marginalized populations; however, a measure of trust is valuable only to the extent that it measures those factors that influence trust in these groups. The PoRT scale, developed in partnership with minoritized racial and ethnic communities, incorporates trust dimensions including secrecy, fairness, community benefit, and privacy. The PoRT scores obtained in this study suggest that there is less trust and more distrust of research among Black and Latino individuals. Use of the validated PoRT scale can improve understanding of trust and facilitate more precise assessment of strategies to amplify trust and trustworthiness of research.

## Notes

Supplement 1. **eMethods.** Development and Validation of the Perceptions of Research Trustworthiness Scale **eFigure.** Conceptual Framework for Developing a Measure of Trust in Biomedical Research **eTable 1.** Trust Content Areas and Frequency of Inclusion in Trust Scales **eTable 2.** Confirmatory Factor Analysis of the Perceptions of Research Trustworthiness Scale and Measures of Its Internal Consistency **eTable 3.** Variables and Analysis Methods Chosen for Scale Validation **eTable 4.** Perceptions of Research Trustworthiness Individual Item Scores Compared by Race and Ethnicity
eReferences

Supplement 2.
Data Sharing Statement
