# Supplementary material for: Development and Validation of the Perceptions of Research Trustworthiness Scale to Measure Trust Among Minoritized Racial and Ethnic Groups in Biomedical Research in the US
Source: JAMA Netw Open. 2022 Dec 29;5(12):e2248812. doi: 10.1001/jamanetworkopen.2022.48812 (PMC9856656; doi:10.1001/jamanetworkopen.2022.48812)
Supplement: Supplement 1. — eMethods. Development and Validation of the Perceptions of Research Trustworthiness Scale eFigure. Conceptual Framework for Developing a Measure of Trust in Biomedical Research eTable 1. Trust Content Areas and Frequency of Inclusion in Trust Scales eTable 2. Confirmatory Factor Analysis of the Perceptions of Research Trustworthiness Scale and Measures of Its Internal Consistency eTable 3. Variables and Analysis Methods Chosen for Scale Validation eTable 4. Perceptions of Research Trustworthiness Individual Item Scores Compared by Race and Ethnicity eReferences [file jamanetwopen-e2248812-s001.pdf]

# Supplementary Online Content

Stallings SC, Cunningham-Erves J, Frazier C, et al. Development and validation of the Perceptions of Research Trustworthiness scale to measure trust among minoritized racial and ethnic groups in biomedical research in the US. *JAMA Netw Open*. 2022;5(12):e2248812. doi:10.1001/jamanetworkopen.2022.48812

**eMethods.** Development and Validation of the Perceptions of Research Trustworthiness Scale
**eFigure.** Conceptual Framework for Developing a Measure of Trust in Biomedical Research
**eTable 1.** Trust Content Areas and Frequency of Inclusion in Trust Scales
**eTable 2.** Confirmatory Factor Analysis of the Perceptions of Research Trustworthiness Scale and Measures of Its Internal Consistency
**eTable 3.** Variables and Analysis Methods Chosen for Scale Validation
**eTable 4.** Perceptions of Research Trustworthiness Individual Item Scores Compared by Race and Ethnicity
**eReferences**

This supplementary material has been provided by the authors to give readers additional information about their work.

© 2022 Stallings SC et al. *JAMA Network Open.*

**eMethods.** Development and Validation of the Perceptions of Research Trustworthiness Scale

We used a four-stage process to develop and validate the Perceptions of Research Trustworthiness (PoRT) Scale. The four stages are: 1) content and item generation, 2) item evaluation through mapping and cognitive interviews, 3) pilot testing, and 4) scale validation.

Stage 1: Content and Item Generation

To generate content and items for the scale, we used a multi-step process that included developing a literature-based conceptual framework, conducting focus groups, and iteratively choosing items using feedback of research team members.

Conceptual framework

The conceptual framework developed to guide our scale development was based on analysis of published studies measuring trust in biomedical research and the extensive literature regarding trust as it relates to research engagement or clinical trial participation. We identified existing scales that measure trust in research, including two scales specific to biomedical research[1,2] and the Distrust Index, which includes seven items on trust of physicians, four of which are focused on research.[3]

Our conceptual framework was guided by the following four concepts:

1. The dimensions of trust in biomedical research vary across populations, and trust among groups underrepresented in research is often related to fairness, safety, honesty and communication.

2. Psychosocial and environmental influencers of trust include cultural beliefs, socioeconomic status, educational attainment, personal health status, personal and group experiences with health systems and research, experienced discrimination, and historical biomedical research abuses.

3. Lack of trust in research is more common among groups often underrepresented in research, including racial and ethnic minorities and those with less education.

4. An individual’s level of trust in research influences their confidence in research results, willingness to volunteer as a research participant or support a family member as participant, adherence to research protocols, and willingness to engage in the design or implementation of research as a community stakeholder.

© 2022 Stallings SC et al. *JAMA Network Open.*

**eFigure.** Conceptual Framework for Developing a Measure of Trust in Biomedical Research

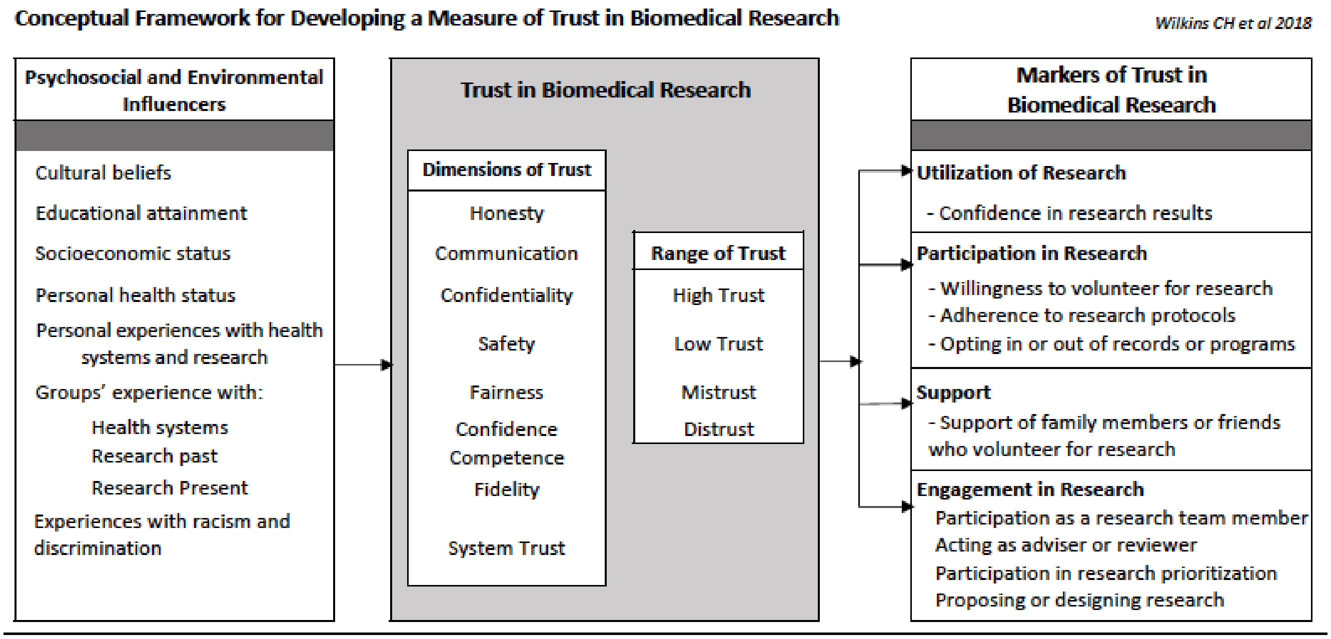


## Literature Review

We conducted a search of the peer-reviewed literature using PubMed, Web of Knowledge and socINDEX™ databases. The search was guided by our conceptual framework and included the following key words: trust, mistrust, distrust, scale, measure, survey, questionnaire and index. Nine trust dimensions have been identified: honesty, communication, fidelity, confidence, competence, confidentiality, global or system trust, safety and fairness. The frequency of use of these dimensions in instruments designed to measure trust are shown in eTable 1. The most frequently measured are honesty, confidence, competence and communication. Few trust instruments focus on fairness and safety, which have previously been reported to be more common among minoritized groups.

**eTable 1.** Trust Content Areas and Frequency of Inclusion in Trust Scales

| Trust Content Areas | Definition | Frequency* in all trust instruments |
|---|---|---|
| Honesty** | Level of integrity and openness in a relationship. | 93% |
| Communication** | Quality and nature of information exchange between all involved parties. | 91% |
| Confidence | Belief in the reliability of involved parties. | 91% |
| Competence | Perceived ability to provide services including qualifications and reputation. | 89% |
| Fidelity | Duty of health sector workers to help patients/participants beyond self-interest. | 60% |
| System trust | Belief in institutions, processes and policies of the health system. | 44% |
| Confidentiality | Maintaining privacy of personal information. | 40% |
| Fairness** | Perceived treatment of disadvantaged and vulnerable groups. | 22% |
| Safety** | Perceived consequences of participation. | Not reported |

Table adapted from systematic review of trust by Ozawa & Sripad 2013.[9]
*Frequency captures % of scales that include items of each trust content area. Includes 45 scales measuring trust in health systems, not limited to research.
**Content areas associated with lower trust/higher mistrust in African Americans and Latinos/Hispanics.

Focus groups

We conducted seven, 90-minute focus groups in target populations exploring perceptions and determinants of research, trust, privacy, confidentiality, and research participation.[4] We recruited participants into race/ethnicity-specific groups to increase the construct validity of the feedback. After obtaining informed consent, the group moderator distributed a survey to participants to capture demographics and degree of trust in biomedical research as measured by either the scale developed by Hall and colleagues[1] or by Mainous and colleagues.[2] For each focus group, a facilitator (one of three we trained for this study) asked open-ended questions based on a discussion guide (See Supplementary material) and probed for statements about the dimensions of trust in biomedical research. Thematic analysis was used to identify themes, and saturation was reached when there were no new emerging themes. [4,5] Additional details regarding focus group conduct and analytic approach are included in the Supplemental Information.

© 2022 Stallings SC et al. *JAMA Network Open.*

**Focus Group Results:** A total of 57 white, Black, and Hispanic/Latino adults, 75% female participated in eight focus groups (published elsewhere).[4] Qualitative analysis of the focus group transcripts revealed racial/ethnic differences in conceptions of research and trust in research.[4] Black and Hispanic/Latino participants identified risks, harms, privacy, secrecy, community benefit, and profit incentives as additional concepts related to trust and trustworthiness in research. Those findings, published elsewhere, highlighted that someone's willingness to participate in research is driven in part by their perception of the trustworthiness of researchers and research institutions, and the information they are given about potential research opportunities. From the qualitative analysis results, we revised and updated the initial Conceptual Framework to reflect a fully cross-cultural understanding of trust in biomedical research, which contained 18 trust dimensions.

Stage 2: Scale Item Evaluation Methods: Mapping and Cognitive Interviews

Iterative Item Development (Mapping)

Our Stage 1 process yielded an initial pool of 42 items, including items modified from existing instruments and new items to address dimension gaps identified from focus group results, guided by dimensions of trust in the conceptual framework. We next screened this pool to refine to a more focused item set through a process of expert consensus. Members of the research team critically reviewed the items and iteratively voted on the mapping of possible scale items to one or more of 18 trust dimensions identified from the literature review and focus group data. Items were: 1) removed if redundant or for lack of fit (e.g., "research is morally wrong"), 2) retained to reflect dimensions not covered in previous instruments or the literature, or 3) re-mapped to a different trust dimension. A final meeting was held to ensure to all feedback was incorporated to the best extent possible. The expert consensus process culminated in a scale including 19 trust items. Each item was constructed as a statement (e.g., "Participation in medical research benefits society.") with responses grounded in a Likert five-point scale ranging from 1 = Definitely Disagree to 5 = Definitely Agree.

Cognitive interviews

We next conducted cognitive interviewing to understand the cognitive process respondents use to understand scale items and evaluate possible sources of response error.[6,7] To further evaluate the comprehension and comprehensiveness of the 19 scale items, we conducted cognitive interviews with two groups of 9 community members each in Nashville, TN. Participants were recruited with snowball sampling through community partners in Nashville and

stratified by race (Black, Latino, and White) and education (High School or Less and Some College or More). Trust scale items were presented to participants using either positive or negative wording. We then used a "think aloud" approach for participants to convey their thoughts so we could identify difficult to understand or unclear items. Participants were compensated with $50 grocery store gift cards.

Responses to, and comments about, scale items were summarized and discussed within the research team. Changes in statement formulation (i.e., negative or positive, word choice, literacy level) in response to CI feedback were discussed by the research team until consensus was achieved. This process led to rephrasing and reordering of items and the addition of a set of term definitions to the beginning of the survey for clarity, establishing face validity from the perspectives of both community members and the research team and reducing potential response error.

The 18 cognitive interviews were equally distributed across white (n=6), Black non-Hispanic/Latino (n=6), and Hispanic/Latino (n=6) participants, and both interview sessions were mixed across groups. Cognitive interview data led to suggested rephrasing and reordering of items and a set of term definitions added to the beginning of the scale for clarity.

Stage 3: Pilot Study Methods

We piloted the resulting 19-item scale measuring trust in biomedical research within a survey that also contained demographic questions (age, education level, relationship status, race/ethnicity, language of choice between Spanish and English for those bilingual in those languages, gender, household income), health care utilization questions (health insurance [yes/no], type of health insurance, most used type of medical care organization, use of alternative care providers), and prior participation in medical research (yes/no/unsure). This pilot survey included a total of 42 items and questions and was anticipated to take approximately 35 minutes to complete.

Participants were recruited in-person via collaboration with community partners and also through two different online platforms, ResearchMatch and Cint.[8,9] In-person participants responded via paper surveys and were recruited from US-Mexico border communities in collaboration with University of Texas Health Science Center San Antonio. For Spanish-speaking respondents, a single interpreter translated the scale for them, audibly. They were compensated with $10 grocery gift cards. Online survey participants recruited through ResearchMatch received $10 electronic gift cards. Online survey participants recruited through Cint were compensated by Cint based on their panel participation. Recruitment messages to new possible participants were sent via ResearchMatch or Cint iteratively until recruitment goals were achieved. Online survey responses

© 2022 Stallings SC et al. *JAMA Network Open.*

were collected using REDCap, and paper survey responses were entered by lab personnel into REDCap.[10]

Stage 4: Validation Study

Validation study participants were recruited through two different online platforms, ResearchMatch and Cint.[8,9] Online survey participants recruited through ResearchMatch were entered into a raffle for 1 of 20 $50 electronic gift cards. Online survey participants recruited through Cint were compensated by Cint based on their panel participation. Online survey responses were collected using REDCap.[10]

Validation Study Results – Confirmatory Factor Analysis, Internal Consistency Reliability, and Test-Retest Reliability.

We examined the scale structure and consistency again for replication of the pilot data results in the validation study data. Factor analysis again showed two different subscales in the validation data. These 2 factors explain 42.2% of the total scale variance, and the KMO and Bartlett's tests show sufficient sampling and significance (data not shown). All but four items had a factor loading >0.50 (four items loading >0.4) on one of the factors and the inter-factor correlation is -0.388. The internal consistency of both subscales was strong, with Cronbach's *α* of 0.867 and 0.716 and item-total correlations exceeding 0.6 and 0.8, respectively. Cronbach's alpha item deleted (AID) and item-total correlations (ITC) were computed and support the internal consistency of the Trust and Distrust subscales (Table 2 in Main Text and eTable 2 below).

**eTable 2.** Confirmatory Factor Analysis of the Perceptions of Research Trustworthiness Scale and Measures of Its Internal Consistency

| **Subscales & Trust Statements (numbered as in survey)** | **Mean (SD) with N=532** | **ITC** | **Cronbach's *α* if omitted** | **Cronbach's *α* of subscale** |
|---|---|---|---|---|
| **Trust Subscale** | | | | 0.72 |
| 1. Medical Researchers tell people everything they need to know about being in a research study. | 3.78 (1.03) | 0.472 | 0.681 | |
| 3. Any info about me that I give to medical researchers would be kept confidential. | 4.23 (0.83) | 0.453 | 0.688 | |
| 4. Medical researchers would never give someone something that would hurt them, just to study how it works in people. | 3.41 (1.23) | 0.487 | 0.677 | |
| 7. Participation in medical research benefits society. | 4.64 (0.55) | 0.446 | 0.698 | |
| 9. Medical Researchers usually tell people in a research study about different things they could do to get well. | 3.25 (1.07) | 0.182 | 0.737 | |
| 10. If I had a chance to be in a medical research study, it would be easy for me to decide to join in or not. | 3.58 (1.19) | 0.432 | 0.690 | |

| | | | | |
|---|---|---|---|---|
| 11. Medical Researchers only do research on people who know it is happening. | 4.14 (0.95) | 0.441 | 0.688 | |
| 14. My physician would not ask me to be in a medical research study if he/she thought it would hurt me. | 4.20 (0.92) | 0.392 | 0.696 | |
| 18. If I had a chance to be in a medical research study, I would be sure that participating in medical research would be the best choice for me. | 4.15 (0.87) | 0.357 | 0.702 | |
| **Distrust Subscale** | | | | 0.866 |
| 2. If I had a chance to be in a medical research study, I wouldn't be sure about being in medical research or not. | 2.34 (1.15) | 0.503 | 0.862 | |
| 5. Medical Researchers keep dangerous things that could happen to people in a medical research study secret. | 2.24 (1.13) | 0.657 | 0.846 | |
| 6. Medical Researchers try to hide any mistakes they make in their research studies. | 2.45 (1.05) | 0.661 | 0.846 | |
| 8. Medical research is secretly designed to give diseases to minority groups. | 1.58 (0.88) | 0.640 | 0.850 | |
| 12. Medical Researchers would lie to people to convince them to be in a research study. | 1.93 (0.98) | 0.739 | 0.840 | |
| 13. Medical Researchers are more interested in helping their own careers than helping people be healthy. | 2.12 (0.98) | 0.632 | 0.849 | |
| 15. It is very likely that I, or people like me, will be used as guinea pigs in medical research. | 2.74 (1.27) | 0.418 | 0.873 | |
| 16. Medical researchers will share my personal info with anybody else they want to, even if I don't tell them they can do that. | 1.75 (0.89) | 0.723 | 0.843 | |
| 17. I'm not sure that I have a voice in who can use my medical info. | 2.32 (1.18) | 0.537 | 0.859 | |

Validation approaches and analyses are described in eTable 3. For the Trust subscale, a higher score indicates a higher level of trust. For the Distrust subscale, a higher score indicates a higher level of distrust. Distributions of means (ranging from 1 to 5) for both subscales showed some skew, thus we employed non-parametric analysis methods. Both subscales showed reliability in test-retest analysis ($r_s = 0.75$, $p < 0.001$ for Trust test-retest and 0.78, $p < 0.001$ for Distrust test-retest). Interclass correlations were strong ($r_s = 0.71$ and 0.76 for Trust and Distrust, respectively) as were intraclass correlations ($r_s = 0.75$ and 0.86 for Trust and Distrust, respectively). Neither Trust nor Distrust score varied significantly between the dichotomous education variable (high school diploma or GED and below versus any college and above), but both scores varied with literacy and numeracy scale scores ($r_s = 0.140$ and 0.215, $p < 0.001$ between Trust and means of literacy and numeracy scores, respectively. $r_s = -0.294$ and -0.151, $p < 0.001$ between Distrust and means of literacy and numeracy, respectively).

© 2022 Stallings SC et al. *JAMA Network Open.*

**eTable 3.** Variables and Analysis Methods Chosen for Scale Validation

| Variables | Type | Coding (range) | Analysis |
|---|---|---|---|
| **Dependent** | | | |
| Trust in research subscale | Continuous | Mean Score (1-5) – Higher score means higher degree of trust | Both Spearman Rho and ICC Correlation for test-retest reliability |
| Distrust in research subscale | Continuous | Mean Score (1-5) – higher score means higher degree of distrust | Both Spearman Rho and ICC Correlation for test-retest reliability |
| **Independent** | | | |
| Trust in healthcare scale | Continuous | Mean Score (1-5) – higher score means higher degree of trust | Spearman Rho Correlation |
| Distrust in healthcare scale | Continuous | Mean Score (1-5) – higher score means higher degree of distrust | Spearman Rho Correlation |
| Education Level | Categorical, dichotomous | 1 – High School or GED, Less than High School<br>2 – Some college or more | Wilcoxon-ranked sum |
| Target groups | Categorical | 1 – Black<br>2 – Latino<br>3 – White | Kruskal-Wallis correlation and pairwise analysis |
| Literacy (3 items) | Continuous | Mean Score (1-5) – higher score means higher literacy | Spearman Rho Correlation |
| Numeracy (3 items) | Continuous | Mean Score (1-6) – higher score means higher numeracy | Spearman Rho Correlation |
| Social Desirability Scale (10 items) | Continuous | Mean Score – (1-2) higher score means increased social desirability | Spearman Rho Correlation |
| Prior research participation | Categorical, dichotomous | 1 – No+Unsure<br>2 – Yes | Wilcoxon-ranked sum |

Criterion Validity

Both subscale mean scores varied with literacy and numeracy scale scores. We employed Spearman's rank-order correlations to assess the relationship between Trust or Distrust scores and literacy scale or numeracy scale mean scores. Increased literacy and numeracy correlated with increased Trust and decreased Distrust, as expected, establishing criterion validity of both two subscales. These statistically significant findings included weak positive correlations between literacy or numeracy and Trust ($r_S = 0.14$, $p < .001$ and $r_S = 0.22$, $p < .001$, respectively), and weak negative correlations between literacy or numeracy and Distrust ($r_S = -0.29$, $p < .001$ and $r_S = -0.15$ $p < .001$, respectively). Neither Trust nor Distrust varied significantly with the dichotomous education variable (high school diploma or GED and below versus any college and above), demonstrating this scale’s capacity to be used across different levels of educational attainment.

Convergent and Discriminant Validity

© 2022 Stallings SC et al. *JAMA Network Open.*

To establish convergent and discriminant validity, we compared the subscale scores with prior research participation and with scores on other validated scales: the “Trust and Distrust among African American Men in Healthcare Scale” developed within our group (manuscript in development), and validated measures of literacy, numeracy, and social desirability. Trust and Distrust in research subscales correlated moderately with the trust and distrust in health care organizations and providers measures ($r_s$ = 0.43, $p < 0.001$ and 0.60, $p < 0.001$ for Trust and Distrust, respectively) and also with self-reported prior research participation, demonstrating the subscales’ validity for measuring concepts of trust and distrust themselves and also in the context of biomedical research. Weak, nonsignificant correlations were observed between each subscale and the Marlowe Social Desirability Scale, demonstrating the subscales’ discriminant properties.

Known Groups Validity

We analyzed the subset of responses that grouped into Black, Hispanic/Latino, and white categories without multiple race choices to eliminate any overlap between the groups (N=448, with 140 Black, 85 Hispanic/Latino, and 215 white). A Kruskal-Wallis H test calculated a statistically significant difference in Trust and Distrust mean subscale scores between the different race/ethnicity groups: H = 17.352, $p < 0.001$ and H = 21.597, $p < 0.001$. Trust scores were higher and Distrust scores lower among white participants as compared to both Black and Hispanic/Latino participants. There were no statistically significant differences in individual item scores between Black and Latino participants as shown in eTable 4.

© 2022 Stallings SC et al. *JAMA Network Open.*

**eTable 4.** Perceptions of Research Trustworthiness Individual Item Scores Compared by Race and Ethnicity

| Statements (numbered as in survey) | Black Participants N=140 | SD | Latino Participants N=85 | SD | White Participants N=215 | SD | P value Black vs White | P value Latino vs White | P value Black vs Latino |
|---|---|---|---|---|---|---|---|---|---|
| 1. Medical researchers tell people everything they need to know about being in a research study. | 3.67 | 1.04 | 3.54 | 1.17 | 3.93 | 0.95 | .11 | .02 | .40 |
| 2. If I had a chance to be in a medical research study, I wouldn't be sure about being in medical research or not. | 2.52 | 1.25 | 2.3 | 1.13 | 2.25 | 1.1 | .08 | .71 | .19 |
| 3. Any info about me that I give to medical researchers would be kept confidential. | 4.22 | 0.86 | 4.24 | 0.83 | 4.24 | 0.8 | .57 | .95 | .57 |
| 4. Medical researchers would never give someone something that would hurt them, just to study how it works in people. | 3.2 | 1.26 | 3.36 | 1.26 | 3.55 | 1.19 | .00* | .12 | .66 |
| 5. Medical researchers keep dangerous things that could happen to people in a medical research study secret. | 2.35 | 1.09 | 2.47 | 1.19 | 2.12 | 1.13 | .21 | .09 | .43 |
| 6. Medical researchers try to hide any mistakes they make in their research studies. | 2.58 | 1.04 | 2.53 | 1.12 | 2.37 | 1.03 | .32 | .54 | .77 |
| 7. Participation in medical research benefits society. | 4.49 | 0.6 | 4.61 | 0.56 | 4.72 | 0.5 | .03 | .16 | .72 |
| 8. Medical research is secretly designed to give diseases to minority groups. | 1.92 | 0.92 | 1.73 | 0.95 | 1.36 | 0.75 | .00* | .00* | .27 |
| 9. Medical researchers usually tell people in a research study about different things they could do to get well. | 3.23 | 1.11 | 3.24 | 1.11 | 3.28 | 1.05 | .66 | .68 | .91 |
| 10. If I had a chance to be in a medical research study, it would be easy for me to decide to join in or not. | 4.03 | 0.98 | 4.17 | 0.96 | 4.19 | 0.93 | .06 | .71 | .18 |
| 11. Medical researchers only do research on people who know it is happening. | 3.4 | 1.24 | 3.14 | 1.2 | 3.82 | 1.11 | .24 | .01 | .39 |
| 12. Medical researchers would lie to people to convince them to be in a research study. | 2.18 | 1.06 | 2.07 | 1.12 | 1.78 | 0.88 | .03 | .20 | .55 |
| 13. Medical researchers are more interested in helping their own careers than helping people be healthy. | 2.16 | 0.92 | 2.2 | 1.05 | 2.07 | 0.99 | .47 | .31 | .79 |
| 14. My physician would not ask me to be in a medical research study if he/she thought it would hurt me. | 3.93 | 1.06 | 4.12 | 0.95 | 4.35 | 0.81 | .04 | .25 | .43 |
| 15. It is very likely that I, or people like me, will be used as guinea pigs in medical research. | 2.93 | 1.22 | 3.01 | 1.28 | 2.58 | 1.28 | .17 | .07 | .46 |

© 2022 Stallings SC et al. *JAMA Network Open.*

| | | | | | | | | | |
|---|---|---|---|---|---|---|---|---|---|
| 16. Medical researchers will share my personal info with anybody else they want to, even if I don't tell them they can do that. | 1.97 | 0.95 | 1.84 | 0.89 | 1.6 | 0.83 | .02 | .18 | .39 |
| 17. I'm not sure that I have a voice in who can use my medical information. | 2.39 | 1.23 | 2.38 | 1.23 | 2.27 | 1.15 | .27 | .24 | .95 |
| 18. If I had a chance to be in a medical research study, I would be sure that participating in medical research would be the best choice for me. | 4.16 | 0.86 | 4.13 | 0.82 | 4.24 | 0.8 | .35 | .32 | .62 |
| *P value of .00 represents a P value of <.001. | | | | | | | | | |

© 2022 Stallings SC et al. *JAMA Network Open.*

## eReferences


1. Hall MA, Camacho F, Lawlor JS, Depuy V, Sugarman J, Weinfurt K. Measuring trust in medical researchers. *Med Care*. 2006;44(11):1048-1053. doi:10.1097/01.mlr.0000228023.37087.cb

2. Mainous AG, Smith DW, Geesey ME, Tilley BC. Development of a Measure to Assess Patient Trust in Medical Researchers. *Ann Fam Med*. 2006;4(3):247-252. doi:10.1370/afm.541

3. Corbie-Smith G, Thomas SB, St George DMM. Distrust, race, and research. *Arch Intern Med*. 2002;162(21):2458-2463.

4. Griffith DM, Jaeger EC, Bergner EM, Stallings S, Wilkins CH. Determinants of Trustworthiness to Conduct Medical Research: Findings from Focus Groups Conducted with Racially and Ethnically Diverse Adults. *J Gen Intern Med*. 2020;35(10):2969-2975. doi:10.1007/s11606-020-05868-1

5. Survey Research Center. Guidelines for Best Practice in Cross-Cultural Surveys. Published online 2016. Accessed March 1, 2019. http://www.ccsg.isr.umich.edu/.

6. Collins D. Pretesting survey instruments: an overview of cognitive methods. *Qual Life Res*. 2003;12(3):229-238. doi:10.1023/a:1023254226592

7. Fortune-Greeley AK, Flynn KE, Jeffery DD, et al. Using cognitive interviews to evaluate items for measuring sexual functioning across cancer populations: improvements and remaining challenges. *Qual Life Res*. 2009;18(8):1085-1093. doi:10.1007/s11136-009-9523-x

8. Harris PA, Scott KW, Lebo L, Hassan N, Lightner C, Pulley J. ResearchMatch: a national registry to recruit volunteers for clinical research. *Acad Med*. 2012;87(1):66-73. doi:10.1097/ACM.0b013e31823ab7d2

9. Cint™ Solutions | Leading Market Research Technology. Cint™. Accessed September 16, 2021. https://www.cint.com/market-research-sample-solutions

10. Harris PA, Taylor R, Thielke R, Payne J, Gonzalez N, Conde JG. Research electronic data capture (REDCap)--a metadata-driven methodology and workflow process for providing translational research informatics support. *J Biomed Inform*. 2009;42(2):377-381. doi:10.1016/j.jbi.2008.08.010


© 2022 Stallings SC et al. *JAMA Network Open.*
