# Supplementary material for: Development and Validation of the Perceptions of Research Trustworthiness Scale to Measure Trust Among Minoritized Racial and Ethnic Groups in Biomedical Research in the US
Source: JAMA Netw Open. 2022 Dec 29;5(12):e2248812. doi: 10.1001/jamanetworkopen.2022.48812 (PMC9856656; doi:10.1001/jamanetworkopen.2022.48812)
Supplement: Supplement 2. — Data Sharing Statement [file jamanetwopen-e2248812-s002.pdf]

# Data Sharing Statement

Stallings. Development and Validation of the Perceptions of Research Trustworthiness Scale to Measure Trust Among Minoritized Racial and Ethnic Groups in Biomedical Research in the US. *JAMA Netw Open*. Published December 29, 2022. doi:10.1001/jamanetworkopen.2022.48812

**Data**
**Data available:** Yes
**Data types:** Deidentified participant data
**How to access data:** consuelo.h.wilkins@vumc.org
**When available:** With publication

**Supporting Documents**
**Document types:** None

**Additional Information**
**Who can access the data:** Researchers with experience and expertise in health equity
**Types of analyses:** any purpose
**Mechanisms of data availability:** without investigator support and with signed data access agreement
